# Correlation analysis between ^18^F-fluorodeoxyglucose positron emission tomography and cognitive function in first diagnosed Parkinson’s disease patients

**DOI:** 10.3389/fneur.2023.1195576

**Published:** 2023-06-13

**Authors:** Sun Zhihui, Li Yinghua, Zhao Hongguang, Dai Yuyin, Du Xiaoxiao, Gao Lulu, Li Yi, Fan Kangli, Zhang Ying

**Affiliations:** Department of Neurology, First Hospital of Jilin University, Changchun, China

**Keywords:** Parkinson’s disease, cognitive impairment, ^18^F-FDG PET, glucose metabolism, cognitive domain, newly diagnosed and untreated PD

## Abstract

**Objective:**

Evaluation of the correlation between ^18^F-fluorodeoxyglucose-positron emission tomography (^18^F-FDG PET) and cognitive function in first-diagnosed and untreated Parkinson’s disease (PD) patients.

**Materials and method:**

This cross-sectional study included 84 first diagnosed and untreated PD patients. The individuals were diagnosed by movement disorder experts based on the 2015 MDS Parkinson’s disease diagnostic criteria. The patients also underwent ^18^F-FDG PET scans and clinical feature assessments including the Montreal Cognitive Assessment (MoCA) scale. Glucose metabolism rates were measured in 26 brain regions using region of interest (ROI) and pixel-wise analyses with displayed *Z* scores. The cognitive function was assessed by professionals using the MoCA scale, which covers five cognitive domains. Spearman’s linear correlation and linear regression models were used to compare the correlations between ^18^F-FDG metabolism in each brain region and cognitive domain, using SPSS 25.0 software.

**Result:**

The results indicated a positive correlation between executive function and glucose metabolism in the lateral prefrontal cortex of the left hemisphere (*p* = 0.041). Additionally, a positive correlation between memory function and glucose metabolism in the right precuneus (*p* = 0.014), right lateral occipital cortex (*p* = 0.017), left lateral occipital cortex (*p* = 0.031), left primary visual cortex (*p* = 0.008), and right medial temporal cortex (*p* = 0.046). Further regression analysis showed that for every one-point decrease in the memory score, the glucose metabolism in the right precuneus would decrease by 0.3 (*B* = 0.30, *p* = 0.005), the glucose metabolism in the left primary visual cortex would decrease by 0.25 (*B* = 0.25, *p* = 0.040), the glucose metabolism in the right lateral occipital cortex would decrease by 0.38 (*B* = 0.38, *p* = 0.012), and the glucose metabolism in the left lateral occipital cortex would decrease by 0.32 (*B* = 0.32, *p* = 0.045).

**Conclusion:**

This study indicated that cognitive impairment in PD patients mainly manifests as changes in executive function, visual-spatial function and memory functions, while glucose metabolism mainly decreases in the frontal and posterior cortex. Further analysis shows that executive function is related to glucose metabolism in the left lateral prefrontal cortex. On the other hand, memory ability involves changes in glucose metabolism in a more extensive brain region. This suggests that cognitive function assessment can indirectly reflect the level of glucose metabolism in the relevant brain regions.

## 1. Introduction

Parkinson’s disease (PD) is a degenerative neurological disorder, characterized by the degeneration and loss of dopaminergic neurons in the substantia nigra of the midbrain. This result in reduced dopamine input to the striatum, and typical motor symptoms such as resting tremor, rigidity, bradykinesia, and postural and gait disorder. With the progress of clinical and pathological research, particularly the introduction of the 2003 pathological grading theory (1) providing a basis for a wide range of symptoms in PD, non-motor symptoms of PD have received increasing attention. This include olfactory dysfunction, constipation, sleep disorders, cognitive dysfunction, and autonomic dysfunction that can occur throughout the disease. Currently, the diagnosis of PD relies more on clinical features, and there is still a dearth of a gold standards for diagnosis.

Application of positron emission tomography (PET) using different tracer agents can achieve *in vivo* molecular pathological diagnosis. Dopamine transporter positron emission tomography (DAT-PET) imaging is highly sensitive in testing for presynaptic dopamine neuron functional disorders (2), and molecular imaging showing normal presynaptic dopamine function was listed as one of the exclusion criteria for PD diagnosis in the Movement Disorder Society (MDS) criteria, 2015 (3). Functional abnormalities can help distinguish essential tremor, drug-induced Parkinson’s syndrome, and vascular Parkinson’s syndrome (4), but DAT-PET imaging has limited value in differentiating PD from other atypical Parkinson’s syndromes such as multiple system atrophy (MSA), progressive supranuclear palsy (PSP), and corticobasal degeneration (CBD) (5). Studies have shown that in PD patients, not only is there dopamine dysfunction, but also significant metabolic abnormalities in the brain. Based on this characteristic, scientists have developed ^18^F-FDG PET specifically for identifying and measuring metabolic abnormalities in PD at the systemic level (6). The ^18^F-FDG PET imaging can reflect local glucose metabolism. Based on this, a Parkinson’s disease-related pattern (PDRP) has been discovered, characterized by a relative increase in metabolism in the pallidum, pons, and cerebellum fibers, and a simultaneous decrease in metabolism in the pre-motor and posterior parietal regions (27). There have been successive studies that have found characteristic metabolic patterns for MSA, PSP, CBD, and DLB. Therefore, glucose metabolism patterns can be used to distinguish between PD and MSA, PSP, and corticobasal syndrome (CBS) (7). However, the factors that affect glucose metabolism in PD are not yet fully understood. There are varying degrees of cognitive impairment in PD, including mild cognitive impairment (MCI) and Parkinson’s disease dementia (PDD). Population-based studies have shown that up to 40% of Parkinson’s patients eventually develop dementia (8). The development of cognitive impairment involves non-dopaminergic pathways, and further research is needed to investigate whether it is related to brain glucose metabolism. Research has shown that compared to PD patients with normal cognitive function, PD-MCI and PDD patients exhibit a progressive reduction in metabolism in the frontal and parietal cortices (9, 10), and cognitive dysfunction is involved in changes within PD brain metabolism. This study aims to explore the correlation between ^18^F-glucose metabolism and cognitive function in newly diagnosed and untreated PD patients. Furthermore, we will examine if, the patient’s glucose metabolism status can be predicted by the cognitive level. Different cognitive impairment patterns may provide clues for establishing possible glucose metabolic patterns.

## 2. Materials and methods

### 2.1. Subjects

The participants in this study were 84 newly-diagnosed and untreated PD patients who had undergone an ^18^F-FDG PET scan at the Parkinson’s Disease Clinic of the First Hospital of Jilin University in northeastern China. Hence, PD was diagnosed by movement disorder experts based on the 2015 MDS Parkinson’s disease diagnostic criteria (3). Other organic or psychiatric disorders that may lead to cognitive impairment were excluded, Alzheimer’s disease, Lewy body dementia, severe cerebrovascular disease, and major depression. The participants were evaluated using the MoCA cognitive scale and other clinical features by professional individuals.

### 2.2. Clinical assessments

Clinical assessments were conducted by trained examiners. The patients underwent standardized neurological examinations wherein, the revised Movement Disorder Society Unified Parkinson’s Disease Rating Scale Part III (MDS-UPDRS III), and the Hoehn and Yahr (H&Y) staging were employed to grade motor symptoms. Neuropsychological evaluations were performed using the Montreal Cognitive Assessment (MoCA) scale (11), and the Hamilton Anxiety Scale (HAMA) and Hamilton Depression Scale (HAMD). Among them, MoCA was used to clinically assess the five cognitive domains. The specific details of the MoCA items are as follows. The short-term memory recall task (5 points) involved two learning trials of five nouns, followed by delayed recall after approximately 5 min. Visual spatial abilities were evaluated by replicating a three-dimensional cube (1 point). Multiple aspects of executive function were evaluated through the following tasks: an alternate task adapted from the trail making B task (1 point), a drawing a clock task (3 points), phonemic fluency task (1 point), and two verbal abstraction tasks (2 points). Attention, concentration, and working memory were assessed through multiple tasks such as sustained attention task-detecting targets by tapping (1 point), serial subtraction (3 points), and forward and backward digit span (1 point each). The language was evaluated by a three-item naming task with unfamiliar animals (lion, camel, rhinoceros; 3 points) and repeating two grammatically complex sentences (2 points).

### 2.3. ^18^F-FDG PET imaging process

Before PET scanning, all PD patients were required to fast for at least 6 h but had free access to water for at least 12 h before imaging, the blood glucose levels needed to be less than 8 mmol/L. The PET scans were performed with a Siemens Biograph 16HR PET/CT (Siemens) in 3D mode. Before the emission scan, a low-dose CT (tube voltage 120 kV, tube current 50 mAs, rotational speed 0.5 s/r, FOV 812 mm × 812 mm, 512 × 512 matrix, slice thickness 2 mm) transmission scan was performed for attenuation correction. The brain emission scan was acquired at least 60 min after intravenous injection of 185 MBq of [^18^F] FDG (Sumitomo HM-12, pH 4–8, radioactive purity >95%, radioactive concentration >370 MBq/mL). The PET scan (1-bed positions, FOV 812 mm × 812 mm, 144 × 144 matrix, slice thickness 2 mm) lasted for 10 min. All procedures were carried out on PD patients under standardized circumstances, i.e., in a quiet and dimly lit room with minimal background noise and a resting state with the eyes open.

### 2.4. Image preprocessing analysis

The FDG images are quantitatively analyzed using Cortex ID Suite software. Cortex ID uses the 3D SSP method developed by Dr. S. Minoshima of the University of Washington. This method first achieves correction and centering of the PET volume using 3D rotation correction, i.e., a symmetry algorithm known as a stochastic sign change (SSC) criterion. Second, stereotactic repositioning of the PET volume with the AC-PC lines is achieved using stereotactic realignment, i.e., iterative matching of the patient image set with a standard atlas template generated by averaging the FDG images of 66 healthy volunteers via linear transformation. Third, the PET volume was matched to a standard image set with a uniform voxel size of 2.25 mm, an interpolation of 60 slices, and a matrix size of 128 × 128 by stretching/compressing it and using a thin-slab spline technique. This way done to nonlinearly distort the PET volume in three dimensions simultaneously along the direction of the major neuronal fiber bundles. This reduces regional mismatches in gray matter locations that remained after linear correction and then automatically standardizes the anatomy of the brain. After that, the algorithm finds the maximum FDG uptake value by “inserting” a 13.5 mm long imaginary 3D vector of the brain in the stereotaxic coordinates of the PET volume with about 16,000 pre-defined cortical surface pixels. Subsequently, these were extracted and assigned to the corresponding surface pixels. The generated uptake maps are normalized pixel-by-pixel to the average metabolic activity across the cerebral cortex. The algorithm uses *Z*-score subtraction to compare the patient’s uptake map pixel by pixel with the uptake map of an age-matched average normal brain. The final display shows a metabolic map of 8 directions and 26 brain regions. Eight of this region, include lateral views of the left and right sides, medial views of the left and right sides, anterior view, posterior view, superior view, and inferior view and the 26 brain regions. The 26 brain regions include the left lateral prefrontal lobe, right lateral prefrontal lobe, left medial prefrontal lobe, right medial prefrontal lobe, left sensorimotor, right sensorimotor, left anterior cingulate, right anterior cingulate, left posterior cingulate, right posterior cingulate, left precuneus, right precuneus, left superior parietal lobe, right superior parietal lobe, left inferior parietal lobe, right inferior parietal lobe, left lateral occipital lobe, right lateral occipital lobe, left primary visual cortex, right primary visual cortex, left lateral temporal lobe, right lateral temporal lobe, left mesial temporal lobe, right mesial temporal lobe, cerebellum and pons. This indicated the number of standard deviations in the patient’s FDG uptake values differ from those of the age-matched normal brain. Areas of severe hypometabolism are shown in red.

### 2.5. Statistical analysis

The data were analyzed using SPSS 25.0 software. Normally distributed data were expressed as mean ± standard deviation, while non-normally distributed data were expressed as median and quartile range (IQR) (Tables 1, 2). Spearman correlation analysis was used to examine the correlation between quantitative variables, and variables with *p* < 0.05 were considered significant. After controlling for age, sex, years of education, HAMA, and HAMD, variables with *p* < 0.1 in the correlation analysis were further included in a stepwise linear regression analysis to establish a linear regression model.

**Table 1:** Demographics and clinical characteristics of PD patients.

Characteristic		Total (*n* = 84) median (IQR)
Age		63.5 (23.8)
Gender	Male	38 (45.2)
	Female	46 (54.8)
Age of onset		58.5 (14.7)
Education years		10.5 (6.5)
H&Y stage		2.0 (1.0)
UPDRS III scores		25 (23.5)
Disease duration		4.0 (5.0)
Handedness	Left	3 (3.6)
	Right	81 (96.4)
MoCA		23 (8)
HAMA		9 (9)
HAMD		8 (8)

**Table 2:** Cognitive domain assessment.

Cognitive domain (total scores)	Test items (total scores)	Scores (IQR)
Executive (7)		5 (3)
	TMT-B (1)	1 (1)
	Draw clock (3)	3 (2)
	Semantic fluency (1)	1 (0)
	Abstraction (2)	1 (1)
Visual spatial (1)		0 (1)
	Cube copy (1)	0 (1)
Language (5)		4 (2)
	Naming (3)	3 (1)
	Repeat (2)	1 (2)
Attention (6)		5 (2)
	Digits forward (1)	1 (0)
	Digits backward (1)	1 (0)
	Target detection using tapping (1)	1 (0)
	Serial 7 substraction starting at 100 (3)	2 (1)
Memory (5)		2 (2)
	Delayed recall (5)	2 (2)

(Total execution = 7, total visual-spatial = 1, total verbal = 5, total attention = 6, total memory = 5, TMT-B, the trial making test part B).

## 3. Result

### 3.1. Correlation analysis of PD patients

**Figure 1:**
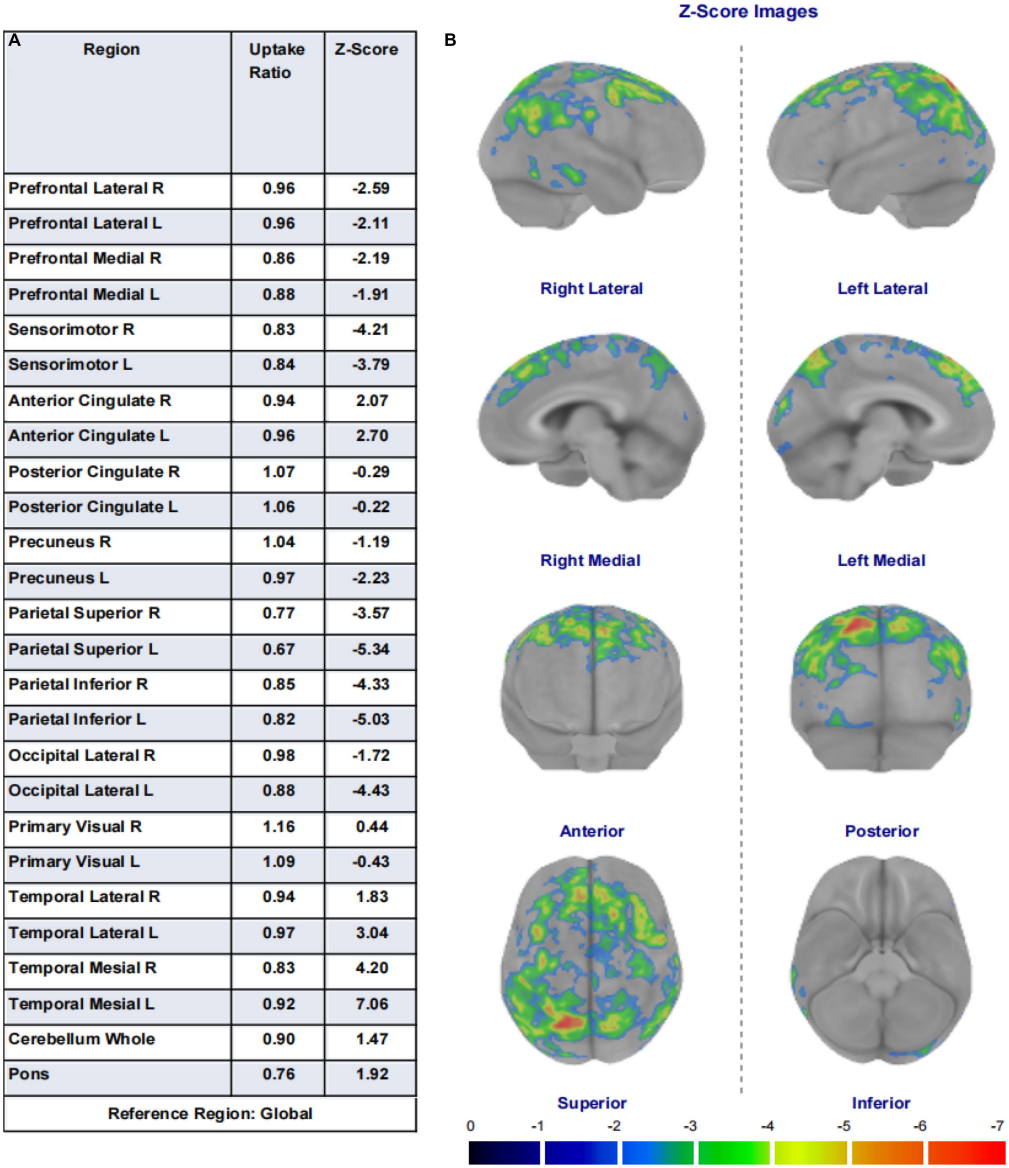
**(A)** Shows the 26 brain regions selected using the region of interest (ROI) method by the Cortex ID Suite program, their respective regional brain glucose uptake rates, and the calculated *Z*-values. **(B)** Are schematic illustrations of the whole-brain glucose metabolism observed in eight directions (lateral views of the left and right sides, medial views of the left and right sides, anterior view, posterior view, superior view, and inferior view). Each unit scale below the image represents a decrease in metabolism of one standard deviation. This image shows brain regions with a decrease in metabolism of two or more standard deviations, with warmer colors indicating greater metabolic deficits.

**Figure 2:**
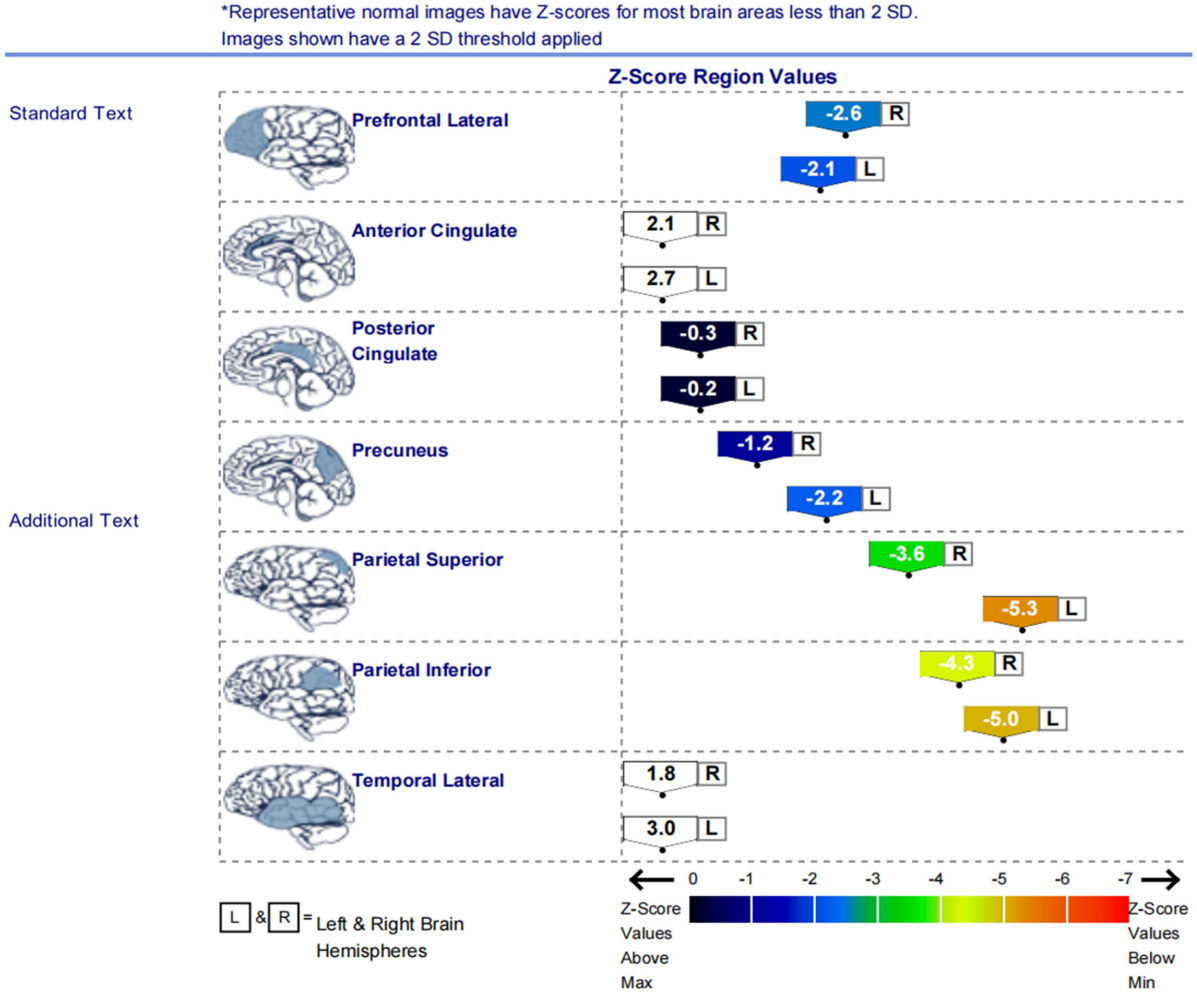
The graph displays the *Z* scores of glucose metabolism in the brain regions of the left and right lateral prefrontal cortex, anterior cingulate cortex, posterior cingulate cortex, precuneus, superior parietal lobule, inferior parietal lobule, and lateral temporal cortex. The low metabolism regions in the patients were mainly located in the lateral prefrontal cortex, precuneus, superior parietal lobule, and inferior parietal lobule on both sides of the brain.

**Figure 3:**
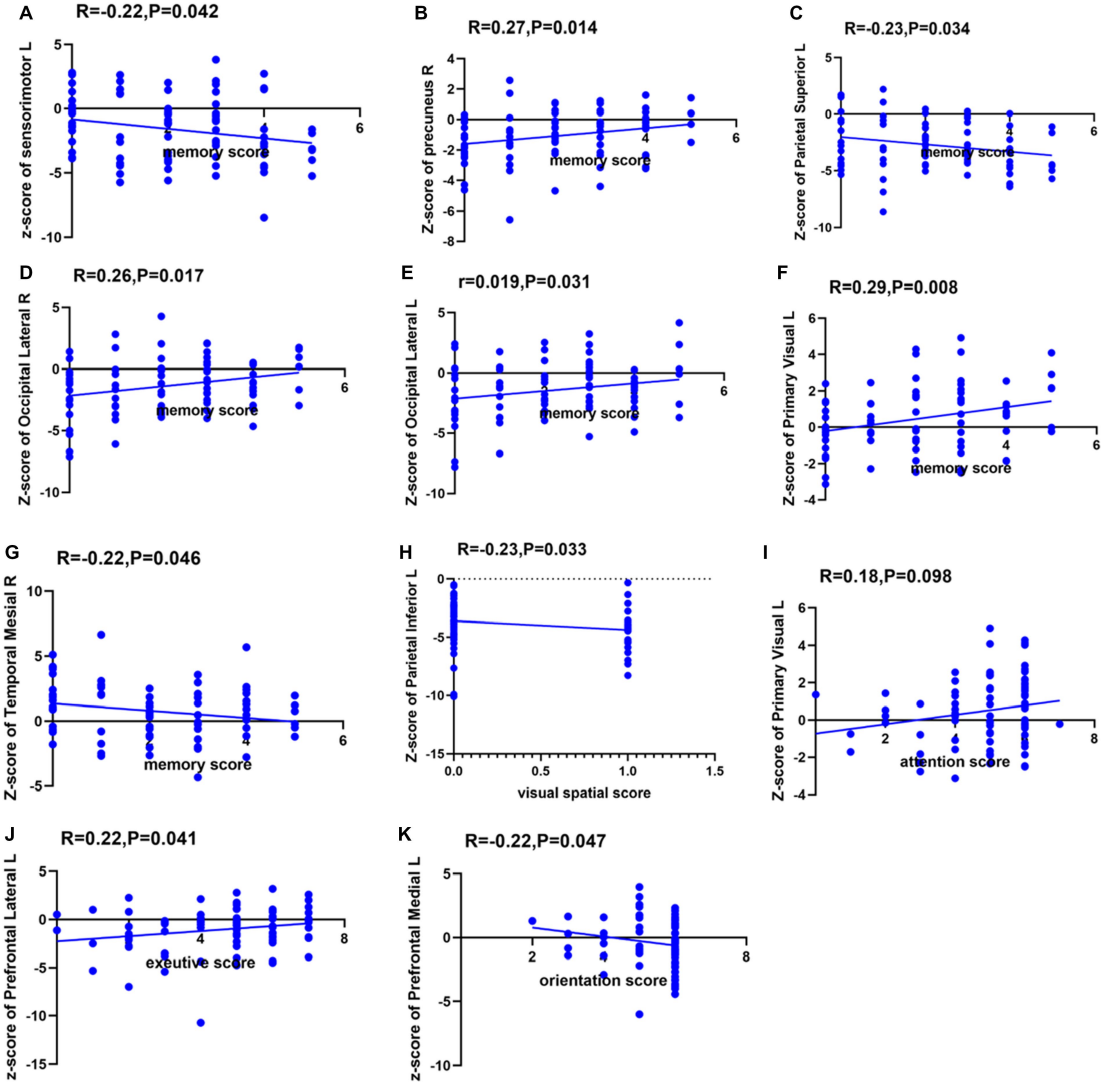
Spearman correlation analysis between glucose metabolism in each brain region and each cognitive domain. The ordinates indicate the glucose metabolism in each brain region, with L indicating the left side and R indicating the right side. **(A)** Spearman correlation analysis between glucose metabolism in left sensorimotor cortex and memory function. **(B)** Spearman correlation analysis between glucose metabolism in right precuneus and memory function. **(C)** Spearman correlation analysis between glucose metabolism in left superior parietal cortex and memory function. **(D)** Spearman correlation analysis between glucose metabolism in right lateral occipital cortex and memory function. **(E)** Spearman correlation analysis between glucose metabolism in left lateral occipital cortex and memory function. **(F)** Spearman correlation analysis between glucose metabolism in left primary visual cortex and memory function. **(G)** Spearman correlation analysis between glucose metabolism in right temporal mesial cortex and memory function. **(H)** Spearman correlation analysis between glucose metabolism in left inferior parietal cortex and visual spatial function. **(I)** Spearman correlation analysis between glucose metabolism in left primary visual cortex and attention function. **(J)** Spearman correlation analysis between glucose metabolism in left lateral prefrontal cortex and executive function. **(K)** Spearman correlation analysis between glucose metabolism in left medial prefrontal cortex and orientation function.

In this study, among the 84 patients, the main glucose metabolism reduction was observed in the prefrontal and posterior cortex, as shown in Figures 1, 2. The left lateral prefrontal cortex was positively correlated with executive function (*p* = 0.041). The left sensorimotor area (*p* = 0.042), the left superior parietal lobule (*p* = 0.034) and right medial temporal cortex (*p* = 0.046) were negatively correlated with memory function. The right precuneus (*p* = 0.014), right lateral occipital cortex (*p* = 0.017), left lateral occipital cortex (*p* = 0.031) and left primary visual cortex (*p* = 0.008) showed were positive correlation with memory function, as shown in Figure 3. The variables with *p* < 0.1 in the Spearman linear correlation analysis were included in the multiple linear regression analysis with each brain region as the outcome variable using the principle of lenient entry and strict exit. A multiple linear regression model was constructed by controlling for age, gender, education, HAMA, and HAMD. The results showed that for every 1-point decrease in memory score, the metabolism in the right sensorimotor area would increase by 0.32 points (*p* = 0.047, *B* = −0.32). Additionally, the metabolism in the left sensorimotor area would increase by 0.37 points (*p* = 0.035, *B* = −0.37), while the metabolism in the right precuneus would decrease by 0.3 points (*p* = 0.005, *B* = 0.30). The metabolism in the left primary visual cortex would decrease by 0.25 points (*p* = 0.040, *B* = 0.25), the metabolism in the right lateral occipital cortex would decrease by 0.38 points (*p* = 0.012, *B* = 0.38) and the metabolism in the left lateral occipital cortex would decrease by 0.32 points (*p* = 0.045, *B* = 0.32). For every 1-point decrease in attention score, the metabolism in the left precuneus would decrease by 0.33 points (*p* = 0.010, *B* = 0.33) (Table 3). To avoid false-positive results, we qualified the *p*-values more strictly and reincorporated the independent variables with *p* < 0.05 into the linear regression analysis. We found that only the left sensorimotor area was negatively correlated with memory function, and positive correlations existed between the right precuneus, bilateral occipital lobe, and the left primary visual cortex and memory function (Supplementary Table S1).

**Table 3:** Spearman correlation analysis of glucose metabolism in each cognitive domain and brain region.

Brain region	Cognitive domain	*p*	*B*	95% CI
Prefrontal lateral R	Executive	0.196	–	–
Prefrontal lateral L	Executive	0.171	–	–
Prefrontal medial L	Memory	0.304	–	–
Sensorimotor R	Memory	0.047	−0.32	−0.64, −0.01
Sensorimotor L	Memory	0.035	−0.37	−0.72, −0.03
Anterior cingulate L	Memory	0.822	–	–
Precuneus R	Memory	0.005	0.30	0.09, 0.51
Precuneus L	Attention	0.010	0.33	0.08, 0.58
Parietal superior L	Memory	0.172	–	–
Parietal inferior L	Visual spital	0.492	–	–
Occipital lateral R	Memory	0.012	0.38	0.09, 0.67
Primary visual R	Memory	0.005	−0.06	−0.09, −0.02
Primary visual L	Attention	0.354	–	–
	Memory	0.040	0.25	0.01, 0.50
Temporal lateral L	Memory	0.385	–	–
Temporal mesial R	Memory	0.223	–	–

## 4. Discussion

This study suggests that the characteristics of cognitive impairment in PD mainly involve executive function, visuospatial function, and memory function, which is consistent with previous research (12). It is also different from AD, which affects memory function, and DLB, which primarily affects visuospatial and attention functions (13, 14). Impairment in executive function is mainly manifested as cognitive flexibility, planning, and impairment of learning ability. That could be attributed to the disruption in the integrity of the dorsolateral prefrontal-striatal loop (dorsolateral prefrontal cortex, dorsolateral striatum, globus pallidus internus, mediodorsal thalamus of the lateral, and posterior parietal cortex) caused by dopamine deficiency (15). The present study found that PD patients with poor executive function showed decreased glucose metabolism in the left lateral prefrontal cortex. The finding is similar to the results of a study by Duncan et al. in 2015 (16) and also consistent with the current theory that executive function is mainly associated with the frontal cortex. In addition, the lateral prefrontal cortex is more sensitive to spatial position information than the amygdala, ventral striatum, and orbitofrontal cortex in the dorsal region of the frontal-striatal loop (17). The clock drawing and the trial making test part B used in this study to assess executive function are also highly correlated with spatial position, suggesting that PD patients may also have some degree of spatial function impairment (18).

Furthermore, this study also observed that in patients with poor memory function, glucose metabolism was reduced in the right precuneus, bilateral occipital cortex, and left visual cortex. Among them, the right anterior cingulate gyrus, bilateral occipital cortex, and left visual cortex displayed positive linear relation to memory function, indicating the lower the metabolic activity of the related cortex, the more severe the memory function impairment. Existing research has shown that memory function is related to the hippocampal-neocortical circuit function, and the neocortex mainly includes the ventromedial prefrontal cortex and posterior cortex, such as the parietal and occipital lobes. The evaluation of memory function in this study used the delayed recall section of the Montreal Cognitive Assessment Scale (immediate recall was not scored). Participants were asked to recall five words they had heard after a 5 min delay and were allowed to receive prompts. Generally speaking, the process of memory retrieval involves two stages: memory construction and memory refinement. The anatomical basis for the construction stage corresponds to the connection between the ventromedial prefrontal cortex and the anterior hippocampus, while the refinement stage involves the connection between the posterior hippocampus and the posterior neocortex, including the occipital and parietal lobes. The neocortex is often involved in tasks such as somatosensory processing, visual recognition, and spatial tasks (19), while the precuneus is closely related to visual spatial representations, situational memory retrieval, etc. This can explain the reduced metabolism in the right precuneus cortex and bilateral occipital cortex found in memory-impaired patients in this study. Another study has also shown that the primary visual cortex is related to memory function (20). Since most of the five words in the MoCA recall task are highly concrete words (such as “red,” “face,” “church,” “velvet,” and “chrysanthemum”), perceptual and storage deficits related to visual information can also affect the formation of memory in patients. Therefore, this can also explain why the memory-impaired patients in this study showed reduced metabolism in the left visual cortices. It is worth noting that this study found that although PD patients have significant memory impairment, their attention function is still relatively intact. This is mainly manifested as impaired short-term and immediate memory, while number-related memory (such as digit span forward and backward) remains relatively intact (see Figure 3).

Interestingly, this study found that patients with poor memory function had increased metabolism in the left medial frontal cortex, left sensorimotor areas, left superior parietal cortex, and right medial temporal cortex. On the other hand, patients with worse visuospatial function had hyper-metabolism in the left superior parietal cortex. Among them, there was a negative linear relation between the bilateral sensorimotor areas and memory function. The presence of high metabolism in certain brain regions in neurodegenerative diseases is a highly controversial topic in the current scenario. First, the neuroinflammation present during disease progression may lead to higher glucose metabolism (21, 22). Second, higher glucose metabolism is a compensatory response to PD-related cortical atrophy with less-damaged brain regions, thereby exhibiting higher glucose metabolism and indicating a decline in cognitive ability. In addition, most of the brain regions with negatively correlated metabolism with cognitive domains belong to the default network. The default network includes the medial prefrontal cortex, medial and lateral parietal cortex, and medial and lateral temporal cortex. Brain regions within the default network exhibit decreased metabolism during task execution. Although patients in this study did not perform cognitive tasks during ^18^F-FDG PET scanning, studies have shown that brain regions with decreased metabolism during novel tasks are not activated during the rest period (23). This suggests that the increased metabolism in the brain regions during PD is related to memory impairment.

This study found that cognitive impairment in PD patients is mainly manifested as changes in executive, visual-spatial and memory function, with decreased glucose metabolism primarily in the frontal and posterior cortex. Among them, the executive ability is related to glucose metabolism in the left lateral prefrontal cortex, while memory ability involves a more extensive range of metabolic changes in the left medial prefrontal cortex, bilateral sensorimotor areas, left primary visual cortex, bilateral occipital cortex, right precuneus, left superior parietal cortex, and right medial temporal cortex. This is broadly consistent with previous studies (24). So, is the pattern of glucose metabolism in the brain of PD patients different from that of other types of cognitive impairment? Vander et al. found that both PDD and Alzheimer’s disease (AD) show a global reduction in glucose metabolism, with similar cortical metabolism in the bilateral parietal, temporal, and frontal lobes (20, 25). When comparing PDD and AD, PDD patients showed a greater decrease in visual cortex metabolism, while metabolism in the medial temporal cortex was relatively preserved (20, 26). In a longitudinal study that lasted for 8 years and used SVM to predict the conversion rate from MCI to PDD, it was found that the SVM-supported PDD classifier was used to classify Lewy body dementia (DLB) patients, with 94.12% of DLB patients predicted to convert to PDD. This suggests that there is no phenotypic difference between PDD and DLB. Therefore, FDG-PET can detect small regional changes in areas such as the frontal lobe, parietal lobe, and occipital lobe at the voxel level and establish a metabolic brain network related to PD cognitive impairment. However, there may still be significant difficulties in using glucose metabolism to differentiate between DLB and PD cognitive impairment.

We believe that this study will help clinicians to predict alterations in glucose metabolism in relevant brain regions by using the patients’ cognitive scores. Additionally, it is also predict possible patterns of glucose metabolism in PD by different impaired cognitive domains to assist in the clinical diagnosis and differential diagnosis of PD.

This study has certain limitations. First, this study adopted a cross-sectional design with a small sample size from a single center. This may have biases in terms of geographic location, race, examination, and evaluation. In the future, we plan to expand the sample size and conduct a longitudinal study with multi-center collaboration to further validate the results. Second, the testing of visual spatial function in this study is relatively limited. We plan to add more tests of visual spatial function to verify the results. Third, we used automatic region of interest (ROI) segmentation, which reduced time and labor costs but was not as accurate as manual delineation. We aim to achieve more accurate, stable, and intelligent automatic sketching and brain partitioning methods in the future.

## Data availability statement

The original contributions presented in the study are included in the article/Supplementary material, further inquiries can be directed to the corresponding author.

## Ethics statement

The studies involving human participants were reviewed and approved by Ethics Committee of First Hospital of Jilin University First Hospital of Jilin University. The patients/participants provided their written informed consent to participate in this study.

## Author contributions

All authors listed have made a substantial, direct, and intellectual contribution to the work and approved it for publication.

## Funding

The work was supported by grants from the National Natural Science Foundation of China (no. 81974194) and the Natural Science Foundation of Jilin Province (no. YDZJ202201ZYTS116) to YZ.

## Conflict of interest

The authors declare that the research was conducted in the absence of any commercial or financial relationships that could be construed as a potential conflict of interest.

## Publisher’s note

All claims expressed in this article are solely those of the authors and do not necessarily represent those of their affiliated organizations, or those of the publisher, the editors and the reviewers. Any product that may be evaluated in this article, or claim that may be made by its manufacturer, is not guaranteed or endorsed by the publisher.
