# Supplementary material for: Correlation analysis between 18F-fluorodeoxyglucose positron emission tomography and cognitive function in first diagnosed Parkinson’s disease patients
Source: Front Neurol. 2023 Jun 13;14:1195576. doi: 10.3389/fneur.2023.1195576 (PMC10294683; doi:10.3389/fneur.2023.1195576)
Supplement: Supplementary file 2 [file Table_2.docx]

**SUPPLEMENTARY TABLE S 2 Linear regression between brain regions and relevant cognitive domains.**

| Brain region |  | Cognitive domain | P | B | 95% CI |
| --- | --- | --- | --- | --- | --- |
| Prefrontal Lateral L | | Executive | 0.171 | - | - |
| Sensorimotor L | | Memory | 0.035 | -0.37 | -0.72, -0.03 |
| Precuneus R | | Memory | 0.005 | 0.30 | 0.09, 0.51 |
| Parietal Superior L | | Memory | 0.172 | - | - |
| Parietal Inferior L | | Visual Spital | 0.493 | - | - |
| Occipital Lateral R | | Memory | 0.012 | 0.38 | 0.09, 0.67 |
| Occipital Lateral L | | Memory | 0.045 | 0.32 | 0.01, 0.63 |
| Primary Visual L | | Memory | 0.040 | 0.25 | 0.01, 0.50 |
| Temporal Mesial R | | Memory | 0.223 | - | - |
